# The activity of antimicrobial peptoids against multidrug-resistant ocular pathogens

**DOI:** 10.1016/j.clae.2024.102124

**Published:** 2024-02-09

**Authors:** Manjulatha Sara, Muhammad Yasir, Parthasarathi Kalaiselvan, Alex Hui, Rajesh Kuppusamy, Naresh Kumar, Sudip Chakraborty, Tsz Tin Yu, Edgar H.H. Wong, Natalia Molchanova, Håvard Jenssen, Jennifer S. Lin, Annelise E. Barron, Mark Willcox

**Affiliations:** a School of Optometry and Vision Science, UNSW Sydney, Australia; b Centre for Ocular Research and Education, University of Waterloo, Canada; c School of Chemistry, UNSW Sydney, Australia; d School of Chemical Engineering, UNSW Sydney, Australia; e The Molecular Foundry, Lawrence Berkeley National Laboratory, Berkeley, CA 4720, USA; f Department of Science and Environment, Roskilde University, 4000 Roskilde, Denmark; g Department of Bioengineering, School of Medicine & School of Engineering, Stanford University, Stanford, CA 9430, USA

**Keywords:** Antimicrobial peptoids, *Pseudomonas aeruginosa*, *Staphylococcus aureus*, Keratitis

## Abstract

**Background:**

Ocular infections caused by antibiotic-resistant pathogens can result in partial or complete vision loss. The development of pan-resistant microbial strains poses a significant challenge for clinicians as there are limited antimicrobial options available. Synthetic peptoids, which are sequence-specific oligo-*N*-substituted glycines, offer potential as alternative antimicrobial agents to target multidrug-resistant bacteria.

**Methods:**

The antimicrobial activity of synthesised peptoids against multidrug-resistant (MDR) ocular pathogens was evaluated using the microbroth dilution method. Hemolytic propensity was assessed using mammalian erythrocytes. Peptoids were also incubated with proteolytic enzymes, after which their minimum inhibitory activity against bacteria was re-evaluated.

**Results:**

Several alkylated and brominated peptoids showed good inhibitory activity against multidrug-resistant *Pseudomonas aeruginosa* strains at concentrations of ≤15 μg mL^−1^ (≤12 μM). Similarly, most brominated compounds inhibited the growth of methicillin-resistant *Staphylococcus aureus* at 1.9 to 15 μg mL^−1^ (12 μM). The *N*-terminally alkylated peptoids caused less toxicity to erythrocytes. The peptoid denoted as TM5 had a high therapeutic index, being non-toxic to either erythrocytes or corneal epithelial cells, even at 15 to 22 times its MIC. Additionally, the peptoids were resistant to protease activity.

**Conclusions:**

Peptoids studied here demonstrated potent activity against various multidrug-resistant ocular pathogens. Their properties make them promising candidates for controlling vision-related morbidity associated with eye infections by antibiotic-resistant strains.

## 1. Introduction

Eye infections, especially microbial keratitis and endophthalmitis, are linked to loss of vision if they can not be appropriately controlled with antibiotics [1,2]. These infections cause approximately 2 million cases of blindness globally [1,2]. In the US alone, microbial keratitis accounts for one million hospital visits and health expenditure of US $175 million a year [3]. In Australia, microbial keratitis costs AU $3 million a year [4]. Contact lens wearers are at 5–10 times increased risk of developing microbial keratitis compared to non-lens wearers, with incidence rates of 4.2 to 13 per 10,000 contact lens wearers per year [4]. Contact lens wear can also predispose people to develop non-infectious keratitis (also called contact lens-induced corneal inflammatory events; CL-CIEs), which is more common than infectious microbial keratitis, with incidence rates in randomised clinical trials of 2 to 6.7 per 100 wearers per year [5].

The most common bacterial causes of microbial keratitis and CL-CIEs are *Staphylococcus aureus, Streptococcus pneumonia*, [6,7] viridians group streptococci and *Pseudomonas aeruginosa*. [8] *Aspergillus* spp., *Fusarium* sp. and *Candida albicans* are common fungal causes of microbial keratitis. [9] Many of these microbes are becoming increasingly resistant to available antimicrobials. Ocular isolates of *P. aeruginosa* have had increased resistance to macrolides and β-lactams for over 30 years during an observation period from 1991 to 2020 [10]. The isolation of methicillin-resistant *S. aureus* from the eye is increasing in occurrence, and often, these strains are also resistant to other commonly prescribed antibiotics [11]. Even though high levels of antibiotics can be applied to the eye, their penetration into the tissue is often low. For example, only 0.15 mg mL^−1^ of ciprofloxacin from 0.3 % (3 mg mL^−1^) eye drops penetrates through the cornea [12]. The concentration that penetrates the cornea is often less than the minimum inhibitory concentration of bacteria isolated from keratitis [13-15]. For example, the MIC for ciprofloxacin of microbial keratitis isolates of *S. aureus* can range from 1 μg mL^−1^ to as high as 2.56 mg mL^−1^ [16] and for isolates of *P. aeruginosa* can range from 0.25 μg mL^−1^ to ≥ 5.12 mg mL^−1^ [17].

A recent outbreak of extensively drug-resistant *P. aeruginosa* keratitis caused by a strain isolated from artificial tears highlights the impact of antimicrobial resistance on ocular infections. As of May 15th’ 2023, 81 patients have been identified from 18 states in the USA with infection from this strain. To date, 14 patients have had vision loss, an additional 4 patients have needed enucleation of the eyeball, and there have been 4 deaths (https://www.cdc.gov/hai/outbreaks/crpa-artificial-tears.html#anchor_1674746879046; accessed 20th May 2023), of which two cases from been published [18]. The strains are of sequence type (ST) 1203 and harbor the *bla*_VIM-80_ and *bla*_GES-9_ genes, and are extensively drug resistant, being resistant to cefepime, ceftazidime, piperacillin-tazobactam, aztreonam, carbapenems, ceftazidime-avibactam, ceftolozane-tazobactam, fluoroquinolones, polymyxins, amikacin, gentamicin, and tobramycin.

The World Health Organisation has recommended development of novel antimicrobial agents to help overcome the infections caused by antibiotic-resistant microbes [19]. Antimicrobial peptides (AMPs) have emerged as potential new antimicrobials [20]. A common mode of action of AMPs, the majority of which are cationic, is via electrostatic interactions with anionic membranes of bacteria [20]. This mode of action often makes it difficult for bacteria to develop resistance as they need to modify the structure of their membranes to do so [21]. The AMP hCAP18/LL-37 is active against *P. aeruginosa* isolated from keratitis [22,23]. The combination of the AMPs melittin and cecropin reduced the pathology of *P aeruginosa* keratitis [24,25]. The AMP melimine has broad spectrum activity against ocular multi-drug resistant bacteria as well as fungi and *Acanthamoeba* sp. [26]. However, despite their promise, naturally occurring AMPs are susceptible to degradation by proteases, are susceptible to changes in pH and salt concentration, and can be toxic towards eukaryotic cells [21].

Several strategies can be employed to enhance stability of peptides such as incorporating β or γ-amino acids, substituting L-amino acids with D-amino acids, or altering side chains from the primary alpha carbon to the backbone amide nitrogen [27,28]. Poly-ε-lysine, made by linking the amino acid via its ε-amino groups rather than α-amino groups, has been formed into bandage contact lenses and combined with penicillin G. This combination had significant antimicrobial activity against *S. aureus* [29]. Poly-ε-lysine bandage lenses combined with amphotericin B had good activity against *C. albicans* [30]. In the current study peptoids are examined which link amino acids via a similar non-conventional method. Peptoids have had their side chains moved from the primary alpha-carbon to an amide, inhibiting backbone chirality [31] and potentially making them resistant to proteolytic cleavage [32]. In addition, these peptoids use alkylated *N*-substituted amino acids to enhance structural resilience making them potent antimicrobial agents that mimic the natural composition of AMPs [31,33].

Peptoids act similarly to AMPs as their cationic regions interact electrostatically with the anionic membranes of bacteria and may also facilitate the targeting of intracellular DNA and ribosomal agglutination [34-37]. They can be more potent than AMPs [31]. For example, peptoids can have minimum inhibitory concentrations as low as 1.8 μg mL^−1^ against *Bacillus subtilis* and 12.4 μg mL^−1^ against *Escherichia coli*, whereas these bacteria had MICs of 4.5 μg mL^−1^ and 35.6 μg mL^−1^, respectively, with the AMP melittin [31] Di-guanidine peptoids, produced via acid amine-coupling between naphthyl-indole amine and with different amino acids were more potent against *S. aureus*, giving MICs of 2.1–6.4 μg mL^−1^ compared to the MIC of ciprofloxacin which ranged from 8 to 256 μg mL^−1^ [38]. Furthermore, peptoids can be active against all the ESKAPE pathogens [39], which are the primary cause of nosocomial (hospital-acquired) infections, as well as bacterial persister cells [40], viruses [41], fungi [40], and parasites [42]. Peptoids also exhibit low immunogenicity akin to AMPs [43,44].

However, a crucial piece of information that remains unexplored is their efficacy against clinical ocular isolates. Therefore, the current study explored the ability of *N*-substituted alkylated glycine peptoids antibacterial activity against ocular microbes. The study also assessed their toxicity and stability to proteases. Understanding variations is crucial for tailoring and optimizing the efficacy of these peptoids against commonly isolated microbes that cause ocular infections and for the further development of peptoids as therapeutic drugs.

## 2. Materials and methods

### 2.1. Microbial strains

All *S. aureus* strains used were isolated from cases of microbial keratitis (Table 1) with various susceptibilities to antibiotics [15,45]. The *S. pneumoniae* strains used were SP04, SP06 and SP07 [46] and *Streptococcus gallolyticus* SV06 (all isolated from eyes of contact lens wearers during adverse events); *P. aeruginosa* PAO1 (which was originally isolated from a wound, and is an invasive strain containing the gene *exoS*) as well as isolates from microbial keratitis, PA 235 (invasive strain containing *exoS*), PA216 (a cytotoxic strain containing *exoU*), PA219 (cytotoxic strain containing *exoU*), PA 233 (cytotoxic strain containing *exoU*), with various susceptibilities to antibiotics were also used [14]. *C. albicans* ATCC 10231, a yeast, isolated from case of a human bronchomycosis, was also evaluated.

### 2.2. Peptoid synthesis

Peptoids were synthesized using the submonomer method with synthetic amines [32]. N-alkylated amines were directly coupled to a solid Rink amide resin and then extended in sequence by reacting primary amines with bromoacetic acid, with alternating condensation of a haloacetic acid and an amine to produce the desired sequence. Once the sequence was achieved, the peptoids were cleaved from the solid support using trifluoracetic acid. Mass spectrometry confirmed compounds’ molecular weights and high performance liquid chromatography of the resulting peptoids showed they were over 97 % pure. The peptoids were chosen as they represent different structures of different sequence length (Table 2). TM1 had the longest overall length, has a helical secondary structure in association with anionic lipid micelles, and forms mostly dimers [39]. TM4 forms helical tetramer bundles and TM14 has an extra lysine mimic group compared to TM4 [39]. TM5, a relatively small alkylated lipopeptoid, forms ellipsoidal micellar assemblies [39]. TM9 contains bromine and an *N*-decyl amino-terminal tail, which assembled into mixtures of ellipsoids and longer worm-like micelles [39]. TM19 was synthesised to contain an alkyl chain but with the addition of the extra lysine mimic group compared to TM9. TM18 was synthesised to be similar to TM19 but without bromine.

### 2.3. Measurement of minimum inhibitory and bactericidal concentrations of peptoids

The peptoids’ minimum inhibitory and bactericidal concentrations (MIC and MBC) were determined using micro broth dilution methods as previously described with slight modifications, [26,39,49,50], and without using acetic acid and bovine serum albumin [39]. Briefly, bacteria or yeast were suspended in Muller Hinton broth (MHB, Oxoid, Thermo Fisher Scientific, Thebarton, SA, Australia) at 0.1 OD_660_nm, which was equivalent to 1x10^8^ colony forming units (CFU) mL^−1^. This suspension was further diluted to achieve 5x10^6^ CFU mL^−1^. The peptoids were diluted in MHB from 250 to 0.24 μg mL^−1^ in 96-well polystyrene microplates (COSTAR, Corning Incorporated, New York, NY, USA) [39]. This range was selected to cover the range of MIC values previously reported for ESKAPE pathogens [39]. This was followed by addition of 100 μL of each microbial suspensions. Wells with only bacteria or the yeast were treated as a negative control, and wells with only MHB medium were treated as a blank. The plates were incubated at 37 °C with shaking at 120 rpm for 24 h. After incubation, the media in each well was serially diluted in phosphate buffered solution and then inoculated onto tryptone soya agar (Oxoid) plates for bacteria, or Sabouraud’s dextrose agar for the yeast, and incubated at 37 °C for 18 hrs. Post incubation, the number of CFUs on the agar plates was counted. The lowest concentration of the peptoids that led to a ≥90 % reduction in the number of CFUs compared to the negative control group without antimicrobial agents was assigned the MIC, while the lowest concentration of the peptoids that led to a ≥99.99 % reduction in the number of CFUs compared to the negative control group was assigned the MBC.

### 2.4. In vitro assay for hemolysis

Hemolysis caused by the peptoids was measured using 18 to 20 mL of a horse or human blood collected in EDTA-coated tubes which has been centrifuged at 500×*g* for 5 min. The supernatant was discarded and the pellet containing the cells were resuspended in PBS to a final volume of 20 mL. This step was repeated five times. After the final wash, the pellet was resuspended in PBS at the ratio of 1:10 (1 mL red blood cell: 9 mL PBS) to yield a final red blood cell (RBC) concentration of approximately 5 × 10^8^ cells mL^−1^. The concentration of RBCs was confirmed using a hemocytometer. The peptoids (stock concentration 2 mM) were diluted sequentially (two-fold dilutions) in PBS to final concentrations of 0.9 μg mL^−1^. All the dilutions were subsequently mixed with the RBC suspension at the ratio of 1:1. MilliQ water was used as a positive control and PBS was used as a negative control in this assay. All samples were incubated at 37 °C for 3 h. Following incubation, the tubes were centrifuged at 500 x *g* for five mins, the supernatants were collected, transferred to wells in a 96 well plate and their optical density was measured at 540 nm [49]. The percentage of hemolysis was calculated by dividing the absorbance of the test sample by the absorbance of the positive control, and multiplying by 100. Data are presented as the hemolysis caused by each dilution of peptoid, and as the concentration of peptoid that caused 10 % or 50 % lysis of red blood cells.

### 2.5. Cytotoxicity

**Fig. 1.**
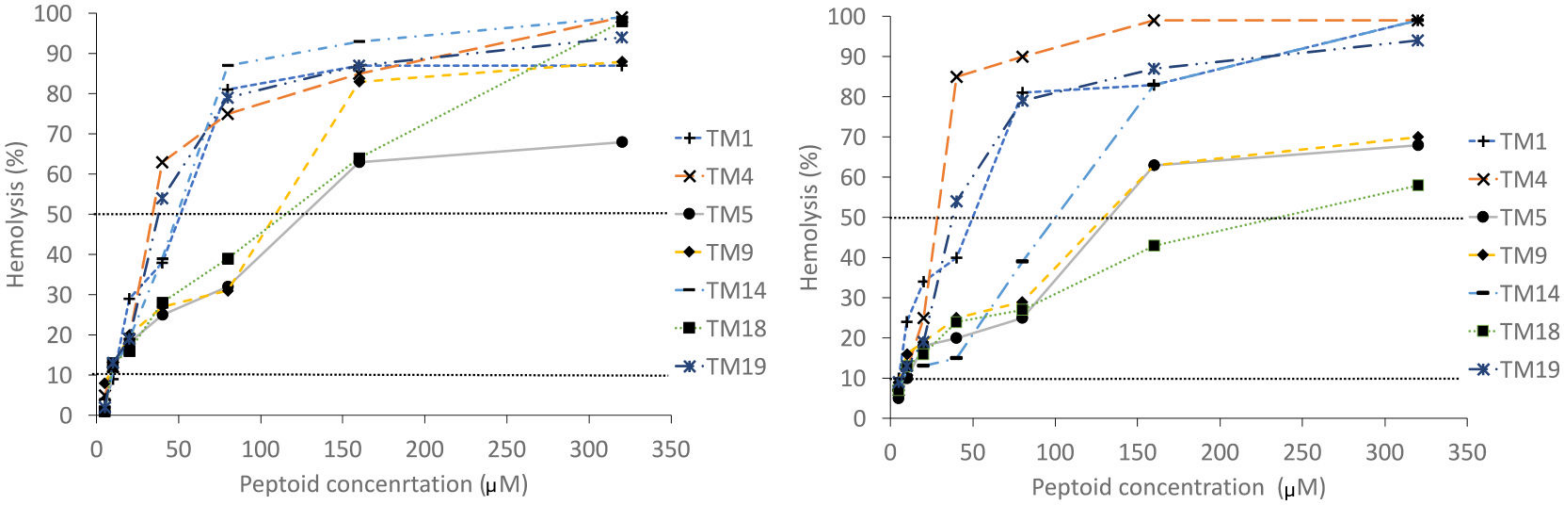
Hemolysis of peptoids with horse (A) and human (B) erythrocytes. The dotted lines represent the concentrations of peptoids that gave 10% (HC_10_) and 50% (HC_50_) hemolysis for each red blood cell type.

Cytotoxicity to corneal epithelial cells was assessed for peptoids TM1, TM5 and TM9 using previously published methods and FDA guidelines [23]. These peptides represent structurally diverse types (Table 2) and had different abilities to cause hemolysis of red blood cells (Fig. 1 and Table 3). Corneal cells were seeded on 96 well plate (GreinerBio One, Frickenhauser, Germany) incubated in 5 % CO_2_ at 37°C and grown to 80 % confluence in Dulbecco’s modified Eagle’s medium (DMEM) (Sigma-Aldrich, Irvine, UK). They were then exposed to twofold dilutions of each peptoid at concentrations ranging from 500 to 1.95 μg mL^−1^ for 24 h at 37°C. An MTT (3-(4,5-dimethylthiazol-2yl)-2,5-diphenyltetrazolium bromide; Sigma-Aldrich) working solution of 0.5 mg mL^−1^ was dissolved in DMEM, 100–200 μL was dispensed into each well and incubated for 4 h at 37°C. Following incubation, MTT was replaced by dimethyl sulfoxide (DMSO, Sigma Aldrich) to dissolve the formazan crystals formed by viable cells. Absorbance of this solution was measured at OD_570nm_. The absorbance is directly proportional to the number of viable cells. The normalized absorbance was calculated to determine number of viable cells using the following formula:

$$\%\text{Cytotoxicity}=\frac{\left(\text{Absorbance of experiment well})-\text{mean}\;(\text{absorbance of control well}\right)}{\text{mean}\;(\text{Absorbance of positive control well})}⋆100$$

Data are also presented as the concentration of peptoids that caused 10 % or 50 % cytotoxicity to human corneal epithelial cells.

### 2.6. Therapeutic index and the selective ratio

The safety of the compounds was estimated by calculating the therapeutic index [51,52], which is the ratio of the concentration of peptoid that caused 50 % hemolysis and the geometric MIC mean, which signifies the central tendency of the MIC of the tested bacteria. The geometric mean is the nth root of the multiplied numbers, where n is the total number of data values. A similar formula was used to calculate the selectivity ratio, but by using the concentration of peptoid that caused 10 % hemolysis [31].

### 2.7. Digestion by proteases

Whilst it is likely that these peptoids, which are non-natural synthetic compounds, would not be cleaved by proteases based on a previous study [53], this had not been experimentally determined before for these particular peptoid designs. Melimine was used as positive control as it is a cationic antimicrobial peptide that can be hydrolysed by proteases. The Expasy peptide cutter module (https://web.expasy.org/peptide_cutter/) was accessed to predict which proteases might digest melimine. The susceptibility of the antimicrobial compounds to digestion by two proteases expected to digest melimine, Proteinase K (120 unit mL^−1^ in 20 mM Tris HCl, pH 7.4; New England Bio Labs, Australia) and Trypsin (10,350 BAFE units/mg in 50 mM borate buffer, pH 8.5; Thermo Scientific products, Australia), was tested. The peptoids and melimine were incubated with each protease at the ratio (w/w) of 1:100 in PBS (pH 7.4) [54] for 24 h at 37 °C. After incubation, the peptoid/melimine + protease solutions were tested for their activity against *P. aeruginosa* 216 which was grown overnight in TSB at 37 °C. Bacterial cells were washed three times in PBS and then resuspended in MHB OD_660nm_ of 0.1 (equal to 1x10^8^ CFU mL^−1^. The bacterial suspension was then diluted in MHB to a final working concentration of 5x10^6^ cells mL^−1^. The diluted bacterial suspension was subsequently added to the wells containing the peptoids/melimine + protease solution and incubated for 24 h at 37 °C. Peptoids/melimine solutions alone (no proteases) were used as a positive control, while protease solutions without the peptoids/melimine were used as a negative control. Post incubation, to inhibit the activity of the proteases, the bacterial suspensions were heated at 55 °C for 10 min in a water bath. After the heating step, the absorbance of the suspensions was measured at OD_660_nm [55].

## 3. Results

### 3.1. Measurement of minimum inhibitory and bactericidal concentrations of peptoids

The antimicrobial activities (MIC and MBC) of the peptoids against bacteria and the yeast are presented in Table 3.

TM1 had MICs of 4 to 9 μM against *Pseudomonas* strains, except isolate PA235 containing *exoS*, where its MIC was 17 μM. TM4 and TM14, which are structurally similar antibacterial agents (Table 1), had MICs of ≤ 12.7 μM against all strains. Among *S. aureus*, the MIC of TM4 was the lowest at 1.6 μM and TM9 the highest at 12.3 μM. TM9 also had the highest MIC against the streptococci. TM5 and TM9 generally had the highest MIC and MBCs. All tested peptoids had potent anti-*Candida albicans* activity with MICs of 1–4.7 μM.

### 3.2. In vitro assay for hemolysis

Fig. 1 gives the hemolysis data for the peptoids against both horse and human erythrocytes. TM5 was the least hemolytic with horse erythrocytes, and for human erythrocytes TM18 was the least hemolytic.

The concentrations of each compound resulting in 10 % (HC_10_) or 50 % (HC_50_) hemolysis [52] is given in Table 4. TM5, TM9 and TM18 had the highest HC_50_ in horse blood, and TM5 and TM18 had the highest HC_50_ in human blood.

### 3.3. Cytotoxicity

**Fig. 2.**
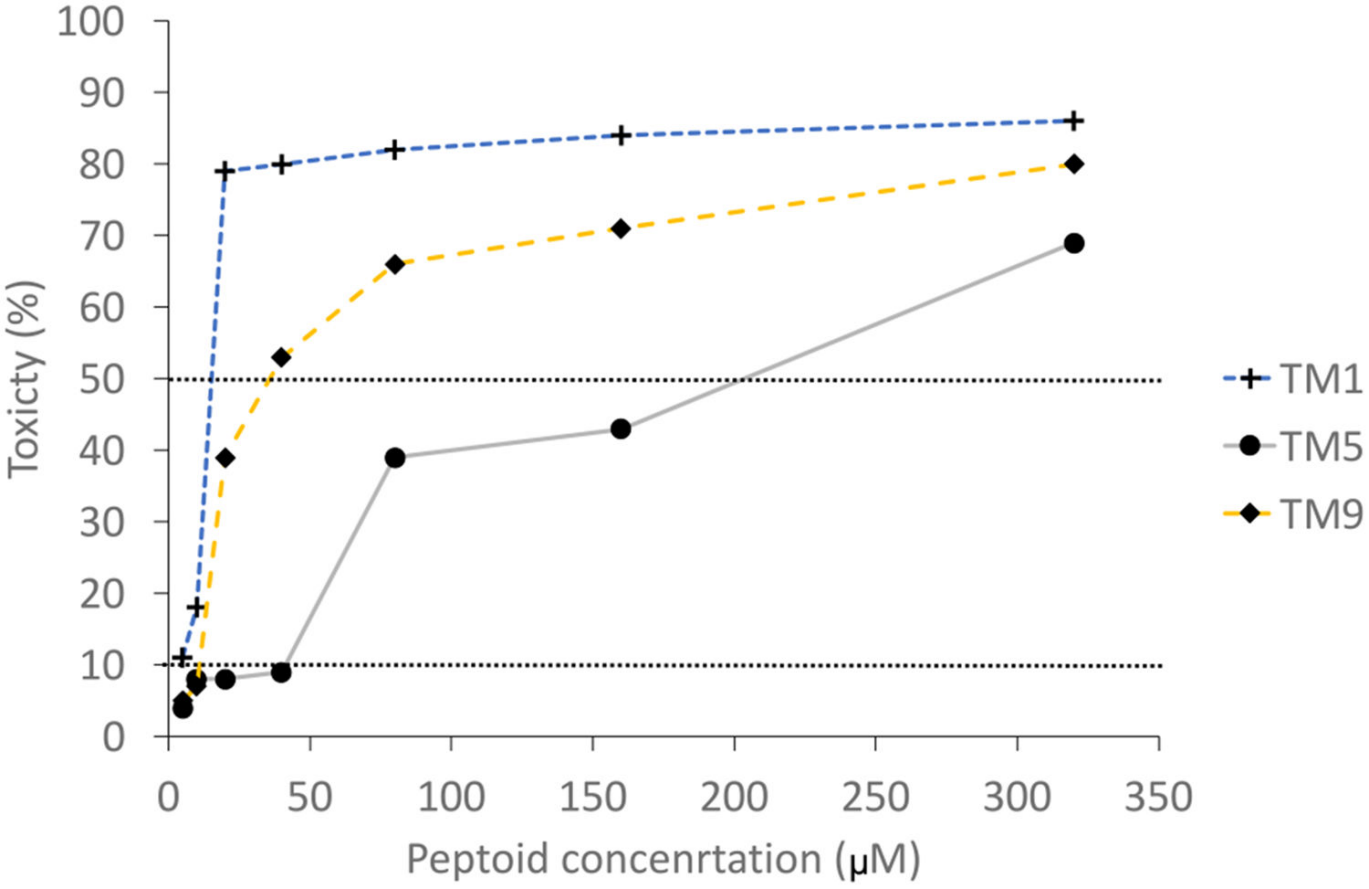
Cytotoxicity of TM1, TM5 and TM9 to human corneal epithelial cells. The dotted lines represent the concentrations of peptoids that gave 10% (CC_10_) and 50% (CC_50_) cell death.

The toxicity to human corneal epithelial cells is given in Fig. 2. TM5 resulted in the lowest levels of cytotoxicity. The concentration of each peptoid that caused 50 % death of the human corneal epithelial cells was 10 μM for TM1, 200 μM for TM5 and 40 μM for TM9 (Table 4).

### 3.4. Therapeutic index and selectivity ratio

A drug’s safety can be expressed as its therapeutic index [31]. The therapeutic index is the ratio of HC_50_ to MIC and the selectivity ratio in the current studies is the ratio of HC_10_ to MIC (Table 4). TM19 showed the lowest therapeutic index and TM5 and TM18 had the highest therapeutic index from the tested series. These findings were similar for the selectivity index.

### 3.5. Digestion by proteases

All the peptoids retained their antimicrobial activity after digestion with either trypsin or proteinase K (Table 5). However, the AMP melimine lost all of its antimicrobial activity when digested with either trypsin or proteinase K (Table 5).

## 4. Discussion

The recent outbreak in the USA of *P. aeruginosa* keratitis caused by extensively drug-resistant strains which was associated with mortality and vision loss has highlighted the need to develop new antimicrobial agents [54]. A class of mimics of AMPs, known as peptoids, have shown promise due to their activity and improved stability compared to peptides. In the current study, the antimicrobial activity of a series of peptoids was tested against susceptible and multidrug resistant strains of ocular pathogens such as *S. aureus*, streptococci, *P. aeruginosa*, and *C. albicans*. These peptoids demonstrated good antimicrobial activity, low toxicity, and good therapeutic indices. These qualities make them ideal for further development as potential therapeutic agents to treat the emerging threat of ocular infections caused by antibiotic resistant microbes.

The activity (MIC) of TM5 against *P. aeruginosa* PAO1 was the same as previously reported [40]. Also, the activity of the current peptoids against *P. aeruginosa* PAO1 was similar to that reported for “peptoid 1” (MIC = 10.8 mM) and “peptoid 2” (MIC = 22.2 mM), although those previously reported peptoids contained *N*-(4-aminobutyl) glycine and aromatic indole groups rather than the *N*-(4-aminobutyl)glycine-(S)-*N*-(1-phenylethyl)glycine aromatic group [34]. Similarly, TM1 and TM5 were active against five antibiotic susceptible or MDR strains of *S. aureus* with MICs of ≤9.4 μM, and five isolates of *P. aeruginosa* with MICs of ≤18.7 μM. This was consistent with previous studies that found the MICs of TM1 and TM5 to be ≤10 μM against multiple ATCC strains of *S. aureus*, and ≤28.0 μM against *P. aeruginosa* [58]. A previous study has reported an MIC of 8.1 μM for TM1 against *C. albicans* SC5314 [31]. The reported MIC is consistent with the MIC against *C. albicans* ATCC 10231 (2.2 μM) found in the current study [31]. Another study reported an MIC of TM1 against *S. pneumoniae* of ≤3.4 μM and the current study yielded similar MIC values against other streptococci (≤8.6 μM) [59]. TM5, which has two lysine mimic side chains, had the greatest antibacterial activity (MIC 4.7 μM) against SA65. This result is consistent with published literature on TM5 [31]. The activity of TM9 was consistent with a previous study with *P. aeruginosa* but not for *S. aureus*. This might be due to the fact only a single strain of each genera was used in the previous study [39]. TM4 has also been investigated in a previous study (named compound 51) [60]. The results were consistent for *S. aureus* (MIC 6.5 μM) and against *P. aeruginosa* (MIC 12 μM), with the current study differing by only one dilution factor. However, the HC_50_ of TM4 in the previous study was 60 μM [60], whereas in the current study the HC_50_ was 25 μM. This is probably due to the use of red blood cells in the current study versus HaCaT cells in the previous study. Indeed, the current study also showed differences in HC_50_ data from erythrocytes and cells in culture.

The chemical difference between TM4 and TM14, whereby TM14 contains an extra lysine mimic group, and hence positive charge, did not greatly affect the overall activity (geometric mean MIC 3.7 vs 4.0 μM) (Table 4) but did improve the haemolytic activity of TM14 (HC_50_ in human blood) compared to TM4 (HC_50_ human blood) by approximately three-fold, as well, consequently, as the therapeutic index (6 vs 23 in human blood). However, for TM9 and TM19 which both contained an alkyl chain, the addition of the extra lysine mimic group to TM19 improved overall antimicrobial activity (24 vs 5; Table 4) but in this case the HC_50_ decreased (135 vs 30 for human blood), resulting in approximately the same therapeutic indices. Another bromine containing peptidomimetic compound with positive charge and lipophilic moieties demonstrated activity similar to TM19, which contains lysine-like side chains and bromines with a decyl alkane chain [43,61]. Short antimicrobial lipopeptides, consisting of four monomers conjugated with a long aliphatic acid, showed broad antimicrobial activity *in vitro* and *in vivo* against human pathogens similar to the current peptoids [62]. The findings suggested that addition of acyl chains, which leads to enhanced antimicrobial activity, rapid killing and reduced toxicity may be due to the lipopeptide’s interaction with the lipid layer.

On the other hand, removing bromine from the structure of TM19, yielding the compound TM18, did not affect antimicrobial activity (Table 4), but greatly improved the HC_50_, giving an improved therapeutic index of 45 with human blood. Various naturally occurring brominated compounds are also antimicrobial, emphasising the probable importance of this addition [63,64]. Therefore, these data indicate that there are balances between charge (from lysine-like side chain) and hydrophobicity (given by alkyl chain or bromine) to obtain optimal antimicrobial activity and safety. This is consistent with the finding that adding bromine to peptoids can improve antimicrobial activity against *S. aureus* and also affects their levels of cytotoxicity [60]. That study demonstrated that the improved activity could be due to the increased the self-assembly of brominated compounds as a result of the increase in hydrophobicity [60]. This is further supported by a study showing how self-assembly of the TM peptoid library correlates with the *in vitro* and *in vivo* antimicrobial activity [39]. A bromine containing peptidomimetic [61] showed similar activity to TM19 in the current study. Another study reported that a fluorinated compound has less hemolysis but increased activity against Gram-positive bacteria [65], again possibly due to changes in the hydrophobicity of the compounds. There appears to be a critical hydrophobicity of compounds beyond which they lose activity, probably due to excessive aggregation [39,60,65].

TM5 was not cytotoxic to corneal epithelial cells at concentrations at least 4-fold higher than its MIC. This further supports that TM5 has a favourable safety profile and confirms TM5 as one of the most promising peptoids for future ocular applications. On the other hand, TM1 and TM9 were cytotoxic to human corneal epithelial cells at or below their MICs. Discrepancies in toxicity compared to hemolysis may be attributed to differences in membrane lipid composition between the cell types with the membrane of RBCs being rich in phosphatidylserine and that of corneal epithelial cells being rich in phosphatidylcholine [66,67]. Moreover, the toxicity assay was performed over 24 h whereas the hemolysis assay was performed for only 3 h, which may also contribute to the observed differences.

The susceptibility of the peptoids to proteolysis was also investigated in a first of its kind study for these TM peptoids. The MICs of the peptoids did not change after exposure to the proteases, confirming the high stability of the peptoids to proteolytic cleavage. The current study demonstrated that peptoids were resistant to the action of two different proteases, trypsin which hydrolyses the peptide bonds at the carboxyl side of lysine or arginine [68,69], and proteinase K which hydrolyses after hydrophobic amino acids [70]. This is important, as one of the major problems with the use of antimicrobial peptides is their degradation by proteases in the body [71]. Therefore, the use of peptoids, for example as a contact lens coating, may overcome the loss of antimicrobial activity that occurs with antimicrobial peptide-coatings [72].

In conclusion, this study has shown that peptoids are active against different bacteria as well as the yeast *C. albicans* that commonly cause ocular infections. The study demonstrated aspects of why different compounds were more active against bacterial cells or less toxic to mammalian cells. Peptoids exhibited potent antimicrobial activity against MDR strains, making them potential antimicrobials for controlling vision loss and morbidity and can help mitigate outbreaks and associated adverse incidents.

## Figures and Tables

**Table 1:** Microbial strains, their source and reported antimicrobial resistance/susceptibility characteristics.

Genera, species and strain number	Source: disease and country	Antibiotic resistance (R)/intermediate susceptibility (I)/susceptibility(S)
*Staphylococcus aureus* 34	MK, Australia	CEFT, AZI, POLYB (R); CIP, GEN, VAN, OXA, CHL (S)[15]
*Staphylococcus aureus* 65	MK, Australia	Not reported
*Staphylococcus aureus* 113	MK, USA	CIP, CEFT, OXA, AZI, POLYB (R); GEN, VAN, CHL (S)[15]
*Staphylococcus aureus* 114	MK, USA	CIP, CEFT, AZI, POLYB (R); GEN, VAN, OXA, CHL (S)[15]
*Staphylococcus aureus* 117	CL-CIE, Australia	CIP, CEFT, AZI, POLYB (R); GEN, VAN, OXA, CHL (S)[47]
*Streptococcus pneumoniae* 04	CL-CIE, India	Not reported
*Streptococcus pneumoniae* 06	CL-CIE, India	Not reported
*Streptococcus pneumoniae* 07	CL-CIE, India	Not reported
*Streptococcus gallolyticus* 04	CL-CIE, India	Not reported
*Streptococcus viridans* 06	CL-CIE, India	Not reported
*Pseudomonas aeruginosa* 01	Skin wound, Australia	CHL, TET (R); CEFT, CIP, TOB (S)[48]
*Pseudomonas aeruginosa* 216	MK, India	CIP, PIP, IMI, CEFT, POLYB (R); LEV, GEN, TOB (S)[17]
*Pseudomonas aeruginosa* 219	MK, India	CIP, LEV, GEN, TOB, PIP, IMI (R); CEFT (I), POLYB (S)[17]
*Pseudomonas aeruginosa* 233	MK, Australia	CIP, CEFT (R); IMI (I), LEV, GEN, TOB, PIP, POLYB (S)[17]
*Pseudomonas aeruginosa* 235	MK, Australia	CIP, PIP, CEFT (R); IMI (I), LEV, GEN, TOB, POLYB (S)[17]
*Candida albicans* ATCC 10231	Bronchomycosis, not reported	Not reported

Abbreviations: MK-microbial keratitis; CL-CIE-contact lens-associated corneal inflammatory events; CEFT = ceftazidime; AZI = azithromycin; POLYB = polymyxin B; CIP = ciprofloxacin; GEN = gentamicin; VAN = vancomycin; OXA = oxacillin; CHL = chloramphenicol; TET = tetracycline; TOB = tobramycin; PIP = piperacillin; IMI = imipenem; LEV = levofloxacin.

**Table 2:** Peptoid sequences and structures.

Compound	Sequence and Molecular weight (MW)	Structure
TM1	H-(*N*Lys-*N*spe-*N*spe)_4_-NH_2_ MW: 1819.36	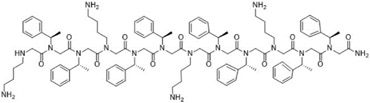
TM4	H-(*N*Lys-*N*spe(p-Br)-*N*spe(p-Br))_2_-NH_2_ MW: 1233.78	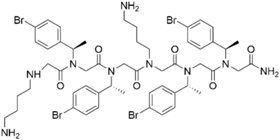
TM5	H-*N*tridec-*N*Lys-*N*spe-*N*spe-*N*Lys-NH_2_ MW: 835.19	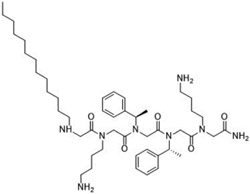
TM9	H-*N*dec-(*N*Lys-*N*spe-*N*spe(p-Br))_2_-NH_2_ MW: 1273.31	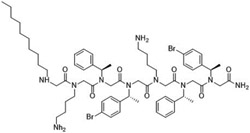
TM14	H-(*N*Lys-*N*spe(p-Br)-*N*spe(p-Br))_2_-*N*Lys-NH_2_ MW: 1357.3	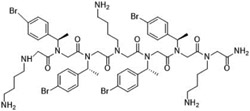
TM18	H-*N*dec-(*N*Lys-*N*spe-*N*spe)_2_- *N*Lys-NH_2_ MW: 1242.8	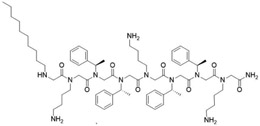
TM19	H-*N*dec-(*N*Lys-*N*spe-*N*spe(p-Br))_2_-*N*Lys-NH_2_ MW: 1398.7	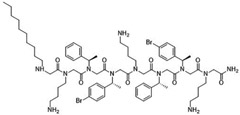

**Table 3:** Antimicrobial activity of peptoids against ocular microbes.

Microbes	TM1	TM4	TM5	TM9	TM14	TM18	TM19
	Minimum inhibitory and bactericidal concentrations μg mL^−1^ (μM)						
	MIC/MBC	MIC/ MBC	MIC/ MBC	MIC/ MBC	MIC/ MBC	MIC/ MBC	MIC/ MBC
***Pseudomonas aeruginosa***							
PAO1	7.8(4.3)/15(8.6)	7.8(6.3)/15(12)	7.8(9.4)/15(18)	31(24)/62(49)	7.8(5.8)/15(11)	15(12)/31(25)	15(11)/15(11)
PA216	15(8.6)/31(17)	7.8(6.3)/31(25)	15(18)/31(37)	31(24)/62(49)	7.8(5.8)/15(11)	15(12)/15(12)	15(11)/31(22)
PA219	15(8.6)/31(17)	7.8(6.3)/15(12)	31(37)/62(74)	31(24)/62(49)	15(11)/15(11)	15(12)/31(25)	31(22)/31(22)
PA233	15(8.6)/15(8.6)	15(12)/15(12)	15(18)/31(37)	31(24)/62(49)	15(11)/31(23)	15(12)/31(25)	7.8(5.6)/15(11)
PA235	31(17)/31(17)	15(12)/15(12)	15(18)/31(37)	31(24)/62(49)	15(11)/31(23)	7.8(6.3)/15(12)	15(11)/31(22)
***Staphylococcus aureus***							
SA34	7.8(4.3)/15(8.6)	1.9(1.6)/1.9(1.6)	7.8(9.4)/7.8(9.4)	15(12)/15(12)	3.9(2.9)/3.9(2.9)	7.8(6.3)/7.8(6.3)	1.9(1.4)/1.9(1.4)
SA65	3.9(2.2)/3.9(2.2)	1.9(1.6)/1.9(1.6)	1.9(2.3)/3.9(4.6)	15(12)/15(12)	1.9(1.4)/1.9(1.4)	3.9(3.1)/3.9(3.1)	1.9(1.4)/1.9(1.4)
SA113	3.9(2.2)/7.8(4.3)	1.9(1.6)/3.9(3.2)	7.8(9.4)/7.8(9.4)	15(12)/31(24)	1.9(1.4)/3.9(2.9)	1.9(1.6)/1.95(1.6)	1.9(1.4)/1.9(1.4)
SA114	7.8(4.3)/7.8(4.3)	1.9(1.6)/1.9(1.6)	7.8(9.4)/7.8(9.4)	15(12)/15(12)	1.9(1.4)/1.9(1.4)	3.9(3.1)/3.9(3.1)	1.9(1.4)/1.9(1.4)
SA117	7.8(4.3)/15(8.6)	1.9(1.6)/1.9(1.6)	7.8(9.4)/7.8(9.4)	15(12)/15(12)	3.9(2.9)/3.9(2.9)	3.9(3.1)/3.9(3.1)	1.9(1.4)/1.9(1.4)
**Streptococci**							
SP04	1.9(1.1)/1.9(1.1)	1.9(1.6)/1.9(1.6)	7.8(9.4) 15(18)	15(12)/31(24)	15(11)/15(15)	15(12)/15(12)	15(11)/15(11)
SP06	3.9(2.2)/7.8(4.3)	1.9(1.6)/1.9(1.6)	1.9(2.3)/1.9(2.3)	62(49)/125(98)	1.9(1.4)/1.9(1.4)	1.9(1.6)/1.9(1.6)	3.9(2.8)/3.9(2.8)
SP07	15(8.6)/15(8.6)	7.8(6.3)/7.8(6.3)	31(37)/31(37)	31(24)/31(24)	15(11)/15(11)	15(12)/15(12)	7.8(5.6)/7.8(5.6)
SG04	3.9(2.2)/7.8(4.3)	7.8(6.3)/15(12)	31(37)/31(37)	62(49)/62(49)	15(11)/15(11)	15(12)/1512)	7.8(5.6)/7.8(5.6)
SV06	3.9(2.2)/7.8(4.3)	7.8(6.3)/15(12)	31(37)/31(37)	62(49)/125(98)	15(11)/15(11)	15(12)/15(12)	15(11)/15(11)
***Candida albicans* (yeast)**							
ATCC 10231	3.9(2.2)/3.9(2.2)	1.9(1.6)/1.9(1.6)	ND	ND	3.9(2.8)/3.9(2.8)	3.9(3.1)/3.9(3.1)	3.9(2.8)/3.9(2.8)

Abbreviations: PA = *Pseudomonas aeruginosa*, SA = *Staphylococcus aureus*, SP = *Streptococcus pneumoniae*, SG = *Streptococcus gallolyticus*, SV = viridans group streptococcus; ND-not determined; MIC = minimum inhibitory concentration; MBC = minimum bactericidal (or fungicidal) concentration [56,57].

**Table 4:** Toxicity of peptoids and their therapeutic index (TI) and selectivity ratio (SR).

Peptoid	Horse HC_10_/ HC_50_ (μM)	Human HC_10_/ HC_50_ (μM)	HCE CC_10_/ CC_50_ (μM)	Geometric mean of MIC (μM)	TI horse blood (ratio HC_50_ to geometric mean MIC)	TI human blood (ratio HC_50_ to geometric mean MIC)	TI HCE (ratio CC_50_ to geometric mean MIC)	SR horse blood (ratio HC_10_ to geometric mean MIC)	SR human blood (ratio HC_10_ to geometric mean MIC)	SR HCE (ratio CC_10_ to geometric mean MIC)
TM1	4/45	8/49	1/10	4	11	12	3	1	2	0.3
TM4	3/25	6/25	ND	4	6	6	ND	0.8	1.5	ND
TM5	5/130	10/140	37/200	9	15	16	22	0.5	1.0	4.1
TM9	3/100	8/135	5/40	24	4	6	2	0.1	0.3	0.2
TM14	3/45	8/90	ND	4	11	23	ND	0.8	2	ND
TM18	3/110	5/225	ND	5	22	45	ND	0.6	1	ND
TM19	3/26	3/30	ND	5	5	6	ND	0.6	0.6	ND

HC_10_/HC_50_ = concentration which caused 10 % or 50 % lysis of red blood cells, CC_10_/CC_50_ = concentration which caused 10 % or 50 % cytotoxicity to human corneal epithelial cells. ND - not determined. HCE = human corneal epithelial cells.

**Table 5:** Antibacterial activity of peptoids against *P aeruginosa* 216 after incubation with proteases.

Peptide/peptoids	MIC μg mL^−1^ (μM)		
	No protease	Proteinase K	Trypsin
Melimine	125 (33)	>250 (66)	>250 (66)
TM4	7.8 (6.3)	7.8 (6.3)	7.8 (6.3)
TM5	15 (18)	15 (18)	15 (18)
TM14	7.8 (5.8)	7.8 (5.8)	7.8 (5.8)
TM18	15 (12)	15 (12)	15 (12)
TM19	15 (11)	15 (11)	15 (11)
